# Supplementary material for: Sex differences in performance and pacing in the greatest Quintuple Iron ultra-triathlon race in history: The IUTA World Championship 2024 in France
Source: PLoS One. 2025 Sep 3;20(9):e0331563. doi: 10.1371/journal.pone.0331563 (PMC12407407; doi:10.1371/journal.pone.0331563)
Supplement: Attachment — Submitted filename: PONE-D-25-29597_Revision 1_Comments.docx [file pone.0331563.s003.docx]

Reviewer #1: General Assessment
This manuscript tackles an ambitious and timely question in ultra-endurance sport by analyzing the largest and fastest Quintuple Iron ultra-triathlon ever held, with 35 finishers (11 women, 24 men). The authors have assembled an impressive dataset to explore how pacing varies across the disciplines (swim, cycling, run) and between sexes. Their findings—especially the record‐breaking female performances—underscore the value of such large‐scale analyses. Although the study has certain limitations (see Limitations section), the insights provided will be of high practical value to athletes, coaches, and exercise scientists investigating human performance in extreme sports.

Major Recommendations
1) Enhance Data Presentation in Figures 1 & 2
Include Raw Data Points: To adhere to the more informative presentation paradigm (Weissgerber et al. 2015), overlay individual data points or violin/box‐and‐scatter combinations behind the bar/line summaries.
Correct Y-Axis Units: Ensure the Y-axis is labeled with the precise unit of time used throughout (e.g., “Time (s)” , as appropriate).
**Answer:** *We agree with the expert reviewer and we included violin with scatter combinations.*

2) p-Values in Table 4
Replace any “0.0” entries in the p-value column with an inequality (e.g., “<0.001”) to avoid implying absolute zero and to conform to standard statistical reporting.
**Answer:** *We agree with the expert reviewer, and we have corrected it.*

3) Streamline and Augment Table 5
Remove Redundant Comparisons: Omit trivial intra-group comparisons (e.g., “Q1_Male vs. Q1_Male”).
Add Effect Sizes: For each relevant pairwise comparison, include columns for the absolute difference (in seconds) and the relative difference (percentage), which will greatly aid practical interpretation.
**Answer:** *We agree with the expert reviewer and corrected reducing the redundances and adding the absolute and relative differences.*

4) Figure 4 Adjustments
Add “(%)” to the Y-axis label for clarity. Apply the same data-presentation rules as for Figures 1 and 2 (raw data points plus summary statistics).
**Answer:** *We believe that the reviewer meant figure 3. We agree with the expert reviewer and we changed figure 3 in agreement with figure 1 and 2.*

Minor Corrections
Line 81: Change the stated run distance from “41.2 km” to the correct “42.2 km.”
**Answer:** *We agree with the expert reviewer and corrected as suggested.*

Practical Implications
Although the authors candidly discuss methodological constraints (e.g., potential selection bias, incomplete split data), their analyses offer actionable guidance. Coaches and athletes can refine training plans and pacing strategies based on discipline- and sex-specific trends, and sport scientists will find this dataset a valuable benchmark for modeling human endurance at the limits.
**Answer:** *We agree with the expert reviewer and included your suggestions in practical applications*

Summary
With the above revisions—particularly improving transparency in figures and tables—the manuscript will achieve a higher level of scientific rigor and practical utility. I look forward to seeing these enhancements in the revised submission.

**Answer:** *We thank the expert reviewer for the comments and hope that our corrections are appropriate.*

Reviewer #2: Manuscript Number: PONE-D-25-29597

Manuscript Title: Sex differences in performance and pacing in the greatest Quintuple Iron ultra-triathlon race in history – the IUTA World Championship 2024 in France

The manuscript is interesting and well written, and well-structured, with several strengths. It represents one of the few studies to utilize detailed data on pacing strategies among ultra-triathletes competing in the fastest and largest Quintuple Iron Ultra-Triathlon World Championship ever held. The study offers novel and valuable insights into sex-based performance dynamics in ultra-endurance sport. The findings suggest that female athletes may benefit from specific endurance strategies, potentially contributing to their competitive advantage in such extreme events. This highlights the importance of developing sex-specific training and pacing approaches to optimize performance in ultra-endurance competitions.

Overall, the different sections of the manuscript are well written. I just have a few minor comments to make.

- The unequal sample sizes between men (n=24) and women (n=11) may influence the statistical power ?. I recommend that the authors explicitly mention this in the limitations section.
**Answer:** *We agree with the expert reviewer and have now explicitly acknowledged in the limitations section that the unequal sample sizes between men (n = 24) and women (n = 11) may influence the statistical power of the comparisons performed. We added ‘Additionally, the unequal sample sizes between men (n = 24) and women (n = 11) may influence the statistical power of sex-based comparisons and should be considered when interpreting the results’.*

- Please use consistent terminology throughout the manuscript. Although "sex differences" and "gender differences" are sometimes used interchangeably, the term "sex" is preferred in scientific sports physiology when referring to biological distinctions.
**Answer:** *We agree with the expert reviewer and used sex. The term gender is now replaced throughout the manuscript.*

- Line 57: I suggest adding the characteristics of the participants (e.g., age).
**Answer:** *We agree with the expert reviewer and included the info.*

- Lines 67 – 37: This paragraph is too long, consider to create small paragraphs.
**Answer:** *We agree with the expert reviewer and we summarized the paragraph.*

- Lines 339 – 340: change “there is a significant interaction was found between sex and performance quartiles in cycling but not in running” to “a significant interaction was found between sex and performance quartiles in cycling but not in running”.

**Answer:** *We agree with the expert reviewer and corrected as suggested.*
